# SpectraNet: a novel model for polyp segmentation leveraging a spectral-guided mixture of functional experts

**DOI:** 10.3389/fonc.2026.1734345

**Published:** 2026-02-18

**Authors:** Zhong Liu, Jing Ling

**Affiliations:** 1 Department of Anorectal Surgery, Jiangyan Hospital Affiliated to Nanjing University of Chinese Medicine, Taizhou, China; 2 Department of Spleen and Stomach Diseases, Jiangyan Hospital Affiliated to Nanjing University of Chinese Medicine, Taizhou, China

**Keywords:** deep learning, foundation model, frequency domain enhancement, mixture of experts, polyp segmentation

## Abstract

Automated and precise polyp segmentation from colonoscopy images is critical for the early diagnosis of colorectal cancer. However, this task is challenged by the ambiguous and low-contrast boundaries of polyps, which often blend with the surrounding mucosa. To address this, we propose SpectraNet, a novel hybrid-domain enhancement network for high-precision polyp segmentation. Our model is built on an encoder-decoder architecture with two core innovations integrated into its skip connections: (1) a Spectral-Guided Boundary Enhancement (SGBE) module that operates in the frequency domain to recover and sharpen indistinct boundary information by enhancing the phase spectrum of features, and (2) a Function-Specialized Mixture-of-Experts (FS-MoE) module that adaptively refines features for diverse polyp morphologies using a set of heterogeneous, function-specific experts. Extensive experiments on our curated PolypSegDataset and two public benchmarks (Kvasir-SEG and CVC-ClinicDB) demonstrate that our method consistently outperforms a wide range of state-of-the-art models. SpectraNet achieves superior performance in key segmentation metrics, and produces qualitatively more accurate segmentation masks with precise boundary definitions.

## 1 Introduction

Colorectal cancer (CRC) poses a significant threat to global health, consistently ranking as one of the leading causes of cancer-related mortality worldwide Center et al. (1) Ladabaum et al. (2). The early detection and removal of adenomatous polyps during colonoscopy remains the most effective strategy for preventing CRC progression Brenner et al. (3) Miller and Knight (4). Central to this preventative measure is the accurate delineation, or segmentation, of polyps, which provides crucial morphological information for clinical assessment Guachi et al. (5). However, manual detection of polyps is a demanding task that is highly time-consuming and dependent on the experience of the clinician, which may lead to significant miss rates, especially for subtle yet clinically important flat or depressed lesions Viscaino et al. (6). To address these limitations, developing robust Computer-Aided Diagnosis (CAD) systems to achieve automated and precise polyp segmentation is of paramount clinical importance for improving diagnosis accuracy and assisting therapeutic decision-making (7).

In response to this need, deep learning-based methods have become the standard for automated polyp segmentation Li et al. (8)Ji et al. (9) Huo et al. (10) Gupta and Mishra (11) Qayoom et al. (12). Architectures based on Convolutional Neural Networks (CNNs), exemplified by the U-Net and its numerous variants, have demonstrated considerable success by learning hierarchical feature representations from images Akbari et al. (13) Yeung et al. (14) Sun et al. (15). More recently, Transformer-based models have been introduced to this domain, leveraging self-attention mechanisms to capture long-range dependencies and global contextual information more effectively than their CNN counterparts Duc et al. (16) Dong et al. (17) Jha et al. (18) Shao et al. (19). Despite variations in architectural design, these state-of-the-art models share a common operational paradigm: they perform inference entirely within the spatial domain, aiming to identify polyp boundaries by learning complex relationships between neighboring pixels and regions.

Despite these advances, the intrinsic visual characteristics of polyps present a persistent challenge Qayoom et al. (12) Mei et al. (20). Lesions frequently exhibit indistinct boundaries, low contrast against the surrounding mucosa, and substantial variation in size, shape, and texture Liu et al. (21) Tajbakhsh et al. (22). However, relying solely on spatial information limits the model’s ability to detect such subtle lesions. In clinical colonoscopy, the visual difference between a flat polyp and healthy mucosa is often negligible. For standard deep learning models (CNNs), which detect objects by looking for sharp changes in pixel intensity (gradients), these low-contrast areas are essentially ‘invisible’. To address this, we look beyond the spatial pixels and advocate for a paradigm shift to the Frequency Domain. In signal processing, an image can be decomposed into its ‘amplitude’ spectrum (intensity energy) and ‘phase’ spectrum (structural information). While the amplitude spectrum largely corresponds to overall contrast and can be susceptible to lighting variations Bracewell (23), the phase spectrum robustly encodes the structural ‘skeleton’ of the object, such as edges and contours Shanmugam et al. (24) Nawab et al. (25). We hypothesize that by explicitly enhancing this phase information, we can recover critical boundary details that are attenuated in the spatial domain, effectively making the ‘invisible’ boundaries visible again.

To this end, we introduce SpectraNet, a novel framework engineered for high-fidelity medical image segmentation. Built upon a frozen vision foundation model and fine-tuned with lightweight, parameter-efficient adapters, SpectraNet’s core innovation lies in a hybrid-domain enhancement unit strategically placed within its skip connections. This unit first employs a Spectral-Guided Boundary Enhancement (SGBE) module to recover critical boundary integrity by operating directly in the frequency domain. Subsequently, a Function-Specialized Mixture-of-Experts (FS-MoE) module performs content-aware feature refinement in the spatial domain to accommodate the vast morphological diversity of polyps.

The main contributions of this paper are summarized as follows:

- We propose SpectraNet for polyp segmentation. It deploys a hybrid-domain enhancement strategy within the skip connections of a parameter-efficiently adapted foundation model. This approach synergistically combines frequency-domain boundary recovery with spatial-domain adaptive refinement to generate highly discriminative multi-scale features.
- We introduce the Spectral-Guided Boundary Enhancement (SGBE) module, a novel component that explicitly enhances the feature phase spectrum to restore high-frequency structural details. This method directly counteracts the inherent limitations of spatial convolutions in detecting low-contrast and ill-defined edges.
- We design the Function-Specialized Mixture-of-Experts (FS-MoE) module, an adaptive mechanism employing a compact set of heterogeneous experts, each meticulously designed for a distinct function (i.e., edge detection, multi-scale texture analysis, and context aggregation). A dynamic gating network routes features for tailored processing, significantly improving the model’s robustness and generalization across diverse polyp morphologies.
- To facilitate more robust evaluation and future research, we introduce PolypSegDataset, a new high-quality benchmark for polyp segmentation. Extensive experiments on this benchmark and several public ones validate that our SpectraNet consistently outperforms previous methods to establish a new state-of-the-art, with its superiority being particularly pronounced on metrics sensitive to fine-grained boundary details.

## 2 Related works

### 2.1 Traditional methods for polyp segmentation

Early research into automated polyp segmentation primarily relied on traditional, hand-crafted feature-based approaches Pogorelov et al. (26). These methods typically targeted low-level visual cues such as color, texture, and shape to distinguish polyps from the surrounding colonic mucosa. Common techniques included color-space analysis, local binary patterns (LBP) for texture description, and edge detection algorithms to identify polyp boundaries Mamonov et al. (27) Maghsoudi (28) Rahim et al. (29). While these methods laid important groundwork, they were often sensitive to variations in illumination, viewpoint, and polyp morphology. Their reliance on manually engineered features limited their generalization capabilities, making it difficult to achieve robust performance across the wide spectrum of polyp appearances seen in clinical practice.

### 2.2 Deep learning methods for polyp segmentation

The advent of deep learning, particularly Convolutional Neural Networks (CNNs) Akbari et al. (13) Brandao et al. (30) He et al. (31)Simonyan and Zisserman (32) Cai et al. (33) Tomar et al. (34) Zhang et al. (35) Sengar et al. (36) Singh and Sengar (37) Khan et al. (38), marked a paradigm shift in medical image segmentation. The U-Net architecture, with its seminal encoder-decoder structure and skip connections, became a foundational model, demonstrating remarkable success in preserving both high-level semantic context and fine-grained spatial details Ronneberger et al. (39) Zhou et al. (40). This spurred the development of numerous variants, such as UNet++ Zhou et al. (40) and ResUNet Zhang et al. (41), which introduced innovations like nested skip pathways and residual connections to further improve feature representation.

More recently, models have been designed specifically to address the unique challenges of polyp segmentation. PraNet Fan et al. (42), for instance, introduced a parallel reverse attention network to explicitly model boundaries and regions, achieving a significant performance leap. Concurrently, the success of Vision Transformers Dosovitskiy et al. (43) in capturing global dependencies led to the development of hybrid models like TransUNet Chen et al. (44) Khan et al. (45), which combines the strengths of both CNNs and Transformers Vaswani et al. (46). The latest research trend involves leveraging large-scale, pre-trained foundation models, such as the Segment Anything Model (SAM) Kirillov et al. (47), as powerful backbones. These models provide robust, generalized feature extraction capabilities that can be fine-tuned for specialized medicaresl tasks like polyp segmentation, representing the current state-of-the-art and the context in which our work is positioned Li et al. (48, 49).

## 3 Methods

### 3.1 Overall framework

**Figure 1:**
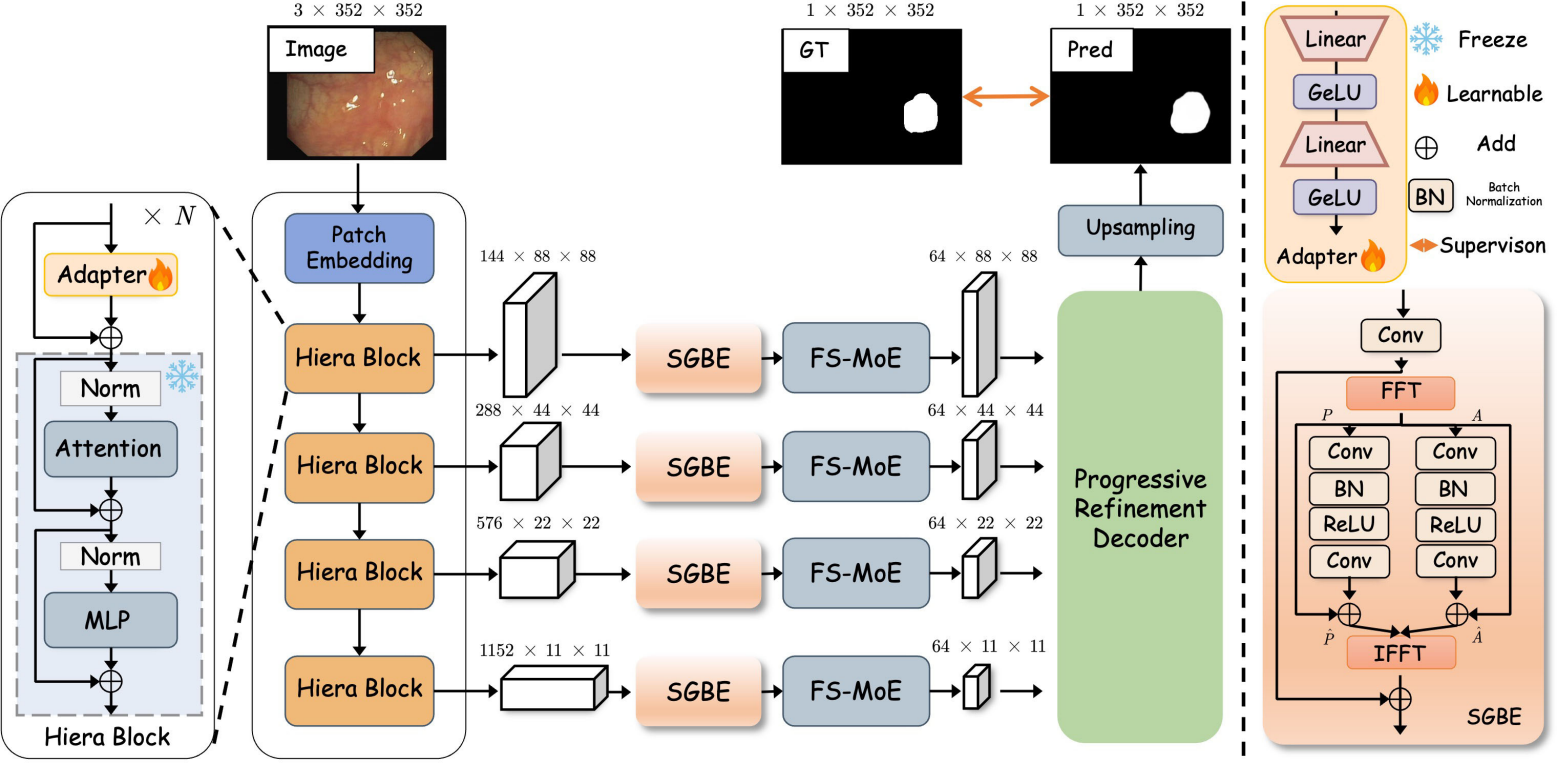
The overall architecture of the proposed SpectraNet. The model is composed of three primary components: (1) a frozen SAM2 encoder enhanced with lightweight trainable adapters for multi-scale feature extraction; (2) a hybrid-domain enhancement unit in the skip connections, featuring a Spectral-Guided Boundary Enhancement (SGBE) module to sharpen boundaries in the frequency domain and a Function-Specialized Mixture-of-Experts (FS-MoE) module for adaptive feature refinement; and (3) a Progressive Refinement Decoder (PRD) that hierarchically fuses the enhanced features, guided by multilevel deep supervision, to produce the final, precise segmentation map.

The overall architecture of our proposed SpectraNet is depicited in **Figure 1**. The network takes a colonoscopy image *I* ∈ ℝ^3×^*^H^*^×^*^W^* as input and produces a pixel-level polyp segmentation probability map *G* ∈ ℝ*^H^*^×^*^W^*. SpectraNet is composed of three primary components: (1) a frozen SAM2 Ravi et al. (50) backbone with a lightweight trainable adapter that extracts robust, multi-scale visual features; (2) a hybrid-domain enhancement unit within the skip connections that first sharpens indistinct boundaries using the Spectral-Guided Boundary Enhancement (SGBE) Module and then adaptively refines features with the Function-Specialized Mixture-of-Experts (FS-MoE) Module; and (3) a progressive refinement decoder that hierarchically fuses the enhanced features from different scales to reconstruct a high-resolution, boundary-precise segmentation mask, supported by multi-level deep supervision.

### 3.2 Encoder

Automated polyp segmentation presents a significant challenge, as the visual characteristics of polyps—particularly flat or early-stage lesions—often lack strong, defining features, blending subtly with the surrounding healthy mucosa. To capture these nuanced patterns, a powerful and robust feature extractor is required. To this end, we employ the Hiera Ryali et al. (51) encoder from SAM2 as our foundational feature extractor. The encoder processes an input image $I\in {\mathbb{R}}^{3\times H\times W}$ and generates a four-level feature pyramid $F_{L}\in {\mathbb{R}}^{B\times H_{L}\times W_{L}\times C_{L}}$ for L={1, 2, 3, 4}. These feature maps have progressively decreasing spatial resolutions (*H_L_* = *H/*2*^L^*^+ 1^, *W_L_* = *W/*2*^L^*^+ 1^) and increasing channel dimensions (*C_L_* = {144, 288, 576, 1152}), capturing a rich hierarchy of representations from fine-grained textures to abstract semantics.

To specialize the powerful, general-purpose features of SAM2 for the medical domain, we introduce a lightweight, trainable adapter into each Hiera block of the encoder, inspired by Houlsby et al. (52) Qiu et al. (53). The adapter, which consists of a linear layer for down-sampling, a GeLU activation function, another linear layer for up-sampling, and a final GeLU activation, processes the feature map *F_L_* to generate a task-specific adaptation vector Δ*F_L_*. This vector is then integrated back into the feature map via a residual connection before the main attention block, producing an adapted feature $F_{L}^{"}$ as defined in Equation 1:

(1) $$F_{L}^{\prime }=F_{L}+\text{Δ}F_{L}$$

This in-place adaptive mechanism allows our model to inject polyp-specific priors directly into the feature extraction hierarchy, effectively steering the general encoder to become a specialized extractor finely tuned for discerning subtle pathological tissues. By keeping the original SAM2 encoder weights frozen and only training the lightweight adapters, we preserve the model’s strong generalized representations while minimizing additional training overhead and reducing the risk of overfitting on smaller medical datasets.

### 3.3 Spectral-guided boundary enhancement module

A primary difficulty in polyp segmentation is the ambiguous and low-contrast nature of polyp boundaries, which challenges convolutional networks that rely on local spatial gradients. However, the structural information that defines these boundaries, while subtle in the spatial domain, is more robustly encoded in the phase component of a signal’s frequency spectrum. Therefore, we designed the SGBE module to operate directly in the frequency domain to amplify this crucial structural information and enhance boundary representation, shown in **Figure 1**.

The module takes the adapted feature map $F_{L}^{\prime }$ from the encoder as input. First, a 1 × 1 convolutional layer projects the features into a uniform channel dimension (*d* = 64), resulting in the feature map *X_L_*. We then transition to the frequency domain by applying a 2D Fast Fourier Transform (FFT). The FFT decomposes the feature map into its amplitude spectrum $A_{L}\in {\mathbb{R}}^{B\times d\times H_{L}\times W_{L}}$ and phase spectrum $P_{L}\in {\mathbb{R}}^{B\times d\times H_{L}\times W_{L}}.$ The amplitude spectrum primarily encodes the energy of spatial frequencies, which corresponds to low-level image statistics like contrast and brightness. The phase spectrum, conversely, is critically important as it preserves the high-frequency structural information that defines the precise spatial location of object boundaries and edges. Both spectra are then passed through parallel enhancement branches, each composed of a sequence of convolutional, BatchNorm, and ReLU layers, to learn enhancement residuals, $\text{Δ}A_{L}$ and $\text{Δ}P_{L}$. These are added element-wise to the original spectra to yield the enhanced versions, ${\hat{A}}_{L}$ and ${\hat{P}}_{L}$, as formulated in Equations 2, 3, respectively:

(2) $${\hat{A}}_{L}=A_{L}+\text{Δ}A_{L}$$

(3) $${\hat{P}}_{L}=P_{L}+\text{Δ}P_{L}$$

The enhanced amplitude and phase spectra are recombined to form an enhanced complex frequency tensor. Subsequently, an Inverse FFT (IFFT) is applied to transform this representation back into the spatial domain, yielding the boundary-enhanced feature map $X_{L}^{\text{enh}}$. To maintain training stability and preserve the original feature context, the final output of the module, $F_{L}^{\text{SGBE}}$, is formed by integrating the enhancement with the input features via a learnable, weighted residual connection as defined in Equation 4:

(4) $$F_{L}^{\text{SGBE}}=X_{L}+\alpha \cdot X_{L}^{\text{enh}}$$

where *α* is a learnable scalar parameter that adaptively controls the contribution of the frequency-domain enhancement. This allows the network to dynamically balance spatial feature fidelity with spectral boundary refinement.

### 3.4 Function-specialized mixture-of-experts module

**Figure 2:**
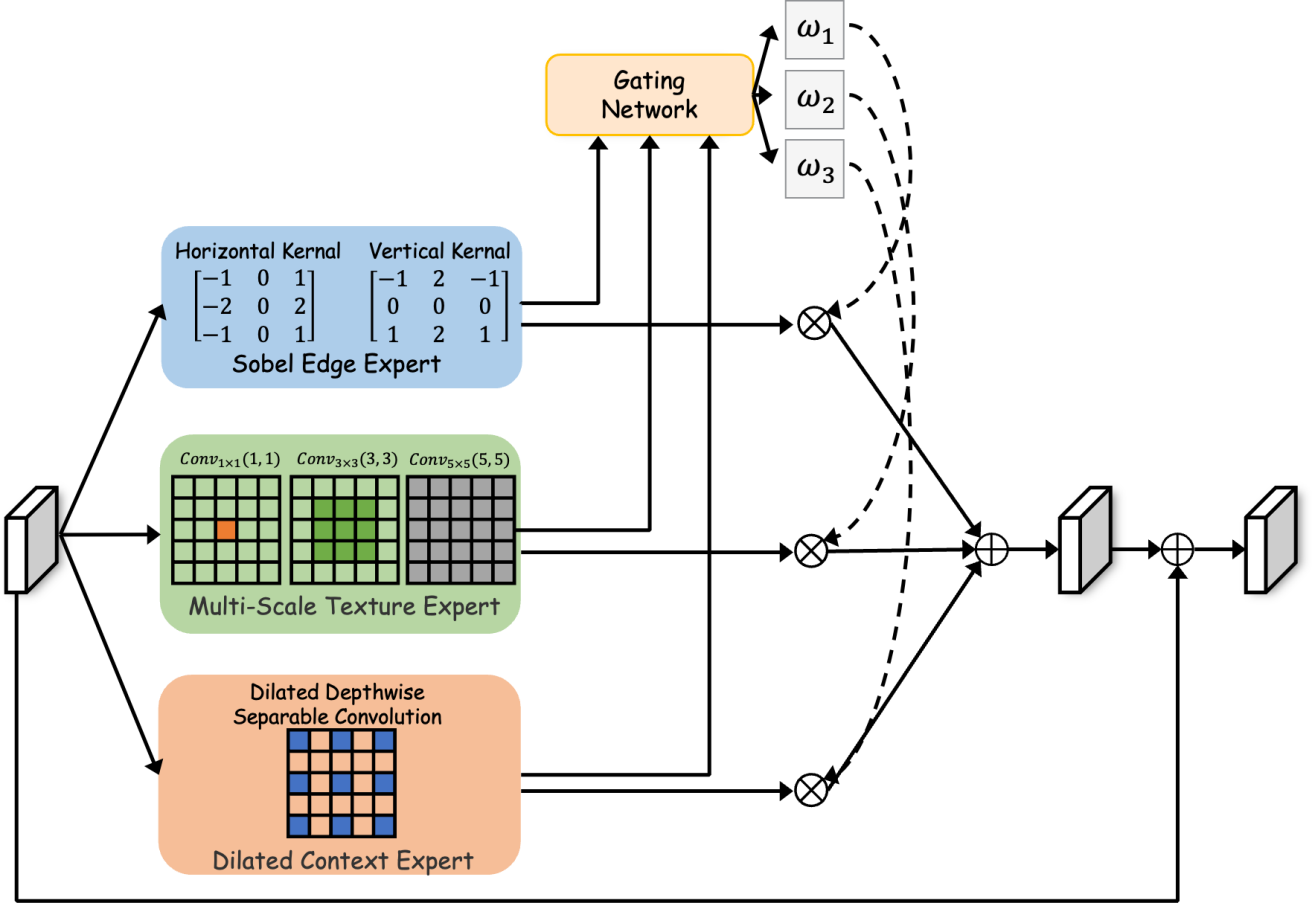
Detailed architecture of the proposed function-specialized mixture-of-experts (FS-MoE) module. An input feature map is processed in parallel by three heterogeneous experts, each designed for a distinct function: (1) a Sobel Edge Expert to enhance high-frequency boundary information, (2) a Multi-Scale Texture Expert to capture varied surface patterns, and (3) a Dilated Context Expert to aggregate broad contextual information. A lightweight gating network dynamically computes weights to adaptively fuse the outputs of the three experts, tailoring the feature refinement for diverse polyp morphologies.

The significant morphological diversity of colorectal polyps, which vary widely in size, shape, and surface texture, demands a highly adaptive feature refinement strategy. To address this challenge, we introduce the Function-Specialized Mixture-of-Experts (FS-MoE) Module. Inspired by the success of MoE designs Zhou et al. (54), our approach utilizes a dynamic routing mechanism to create a content-aware feature processing pipeline. Unlike conventional MoE systems that use homogeneous experts, our FS-MoE employs a compact set of *heterogeneous experts*, where each is meticulously designed for a distinct and complementary function. The module processes the input feature map *F_L_*^SGBE^ through three parallel, function-specialized expert branches – Sobel Edge Expert Branch, Multi-Scale Texture Expert Branch and Dilated Context Expert Branch, which is shown in **Figure 2**.

The Sobel Edge Expert is designed to explicitly capture and enhance high-frequency boundary information. It consists of a lightweight, depthwise 3×3 convolution where the kernels, $K_{sobel}\in {\mathbb{R}}^{d\times 1\times 3\times 3}$, are initialized with classic Sobel operators Kittler (55). This provides a strong inductive bias for edge detection, which is subsequently fine-tuned during training. The operation is formally defined in Equation 5:

(5) $$E_{edge}(F)=\sigma (\text{BN}(F{*}_{dw}K_{sobel}))$$

where ∗*_dw_* denotes the depthwise convolution operation, BN is BatchNorm, and *σ* is the GeLU activation function.

The Multi-Scale Texture Expert aims to capture varied surface patterns at multiple scales. It employs a three-branch parallel design where the input feature *F* is first projected into a low-dimensional space, $F_{mid}={\text{Conv}}_{1\times 1}^{d\to d/8}(F)$. The three branches then operate on $F_{mid}$ to produce outputs *B*_1_*, B*_2_*, B*_3_ with varying receptive fields, as formulated in Equation 6:

(6) $$B_{1}={\text{Conv}}_{1\times 1}(F_{mid})$$

$$B_{2}={\text{Conv}}_{3\times 3}(F_{mid})$$

$$B_{3}={\text{Conv}}_{5\times 5}(F_{mid})$$

As shown in Equation 7, these outputs are concatenated and fused via a final 1 × 1 convolution to restore the original channel dimension *d*, producing a rich, multi-scale texture representation:

(7) $$E_{texture}(F)={\text{Conv}}_{1\times 1}^{d/8\times 3\to d}(\text{Concat}[B_{1},B_{2},B_{3}])$$

To efficiently aggregate broad contextual information, the Dilated Context Expert utilizes a depthwise separable convolution. This two-stage process involves a 3 × 3 depthwise convolution with a large dilation rate (dil = 2), followed by a 1 × 1 pointwise convolution to combine channel features, as expressed in Equation 8:

(8) $$E_{context}(F)={\text{Conv}}_{1\times 1}^{\text{pw}}(\sigma (\text{BN}({\text{Conv}}_{3\times 3}^{\text{dw},\text{ dil}=2}(F))))$$

The core of the module is its adaptive gating mechanism. A lightweight gating network, G(·), processes the input feature *F_L_*^SGBE^ to dynamically generate a set of scalar weights $w\in {\mathbb{R}}^{3}$. As formulated in Equation 9, the refined feature map, $X_{L}^{\text{ref}}$, is obtained by a weighted sum of the expert outputs, $E_{j}(\cdot )$:

(9) $$X_{L}^{\text{ref}}=\sum_{j=1}^{3}w_{j}\cdot E_{j}(F_{L}^{\text{SGBE}})$$

Finally, a residual connection with a learnable scalar parameter, *β*, is applied to form the final output, $F_{L}^{\text{FS}-\text{MoE}}$ according to Equation 10:

(10) $$F_{L}^{\text{FS}-\text{MoE}}=F_{L}^{\text{SGBE}}+\beta \cdot X_{L}^{\text{ref}}$$

This adaptive fusion empowers our network to intelligently tailor its feature refinement strategy, improving its ability to accurately segment polyps across their wide spectrum of visual presentations.

### 3.5 Progressive refinement decoder

Although our enhanced skip connections provide rich, multi-scale feature representations, effectively fusing them to reconstruct a precise segmentation mask is non-trivial. Directly merging features from different scales can lead to coarse boundaries or the dilution of semantic information. To address this, we employ a Progressive Refinement Decoder (PRD), inspired by top-down fusion approaches proven effective in dense prediction tasks. The decoder is designed to hierarchically aggregate the enhanced multi-scale features, progressively recovering fine spatial details while preserving high-level semantic context.

The decoder reconstructs the final prediction through a sequence of refinement stages, beginning with the deepest feature map from the FS-MoE module, $D_{4}=F_{4}^{\text{FS}-\text{MoE}}$. For each subsequent decoding stage *i* ∈ {3, 2, 1}, a Progressive Refinement Module (PRM) integrates the upsampled features from the deeper stage, *D_i_*_+ 1_, with the corresponding enhanced skip connection feature, $F_{i}^{\text{FS}-\text{MoE}}$. This integration process is formally defined in Equation 11:

(11) $$D_{i}={\text{Φ}}_{i}(\text{Concat}[\text{Upsample}(D_{i+1}),F_{i}^{\text{FS}-\text{MoE}}])$$

where Upsample(·) denotes a 2× bilinear upsampling operation and Φ*_i_*(·) is a refinement block composed of a 3 × 3 convolution, BatchNorm, and a GeLU activation. This fusion process yields a series of intermediate decoder features {*D*_3_*, D*_2_*, D*_1_} with progressively increasing spatial resolution.

To guide the learning process at all scales, a prediction head, $H_{i}(\cdot )$, is applied to the output of each decoder stage to produce an auxiliary segmentation map, *out_i_*. Each head consists of a 1 × 1 convolution followed by a sigmoid activation function. These auxiliary maps (*out*_4_*, out*_3_*, out*_2_) are upsampled to the original input resolution and used for deep supervision, as detailed in the following section. The final, primary segmentation map, *out*_1_, is produced from the feature map of the last and highest-resolution decoder stage, *D*_1_.

This progressive, top-down refinement structure ensures that strong semantic guidance from the deeper layers is effectively propagated and fused with the detailed, boundary-rich features at shallower layers. This process enables the network to achieve both a globally consistent understanding of the polyp and pixel-level precision in the final segmentation mask.

### 3.6 Multi-scale supervision

To ensure robust feature learning across all scales, the network is trained using a deep supervision strategy where a loss is applied to the output of each of the four decoder stages. This total loss function, *L*_total_, is formulated in Equation 12 as a weighted sum of the loss from the primary output (*out*_1_) and the three auxiliary outputs (*out*_2_*, out*_3_*, out*_4_):

(12) $$L_{\text{total}}=L_{\text{seg}}(out_{1},G)+\sum_{i=2}^{4}\lambda_{i}\cdot L_{\text{seg}}(\text{Upsample}(out_{i}),G)$$

where *G* is the ground-truth polyp mask and *λ_i_* are loss weights that balance the gradients from different scales. Based on empirical evaluation, we set the weights for the auxiliary outputs to *λ_i_* = 0.1 for *i* ∈ {2, 3, 4}.

Following Dong et al. (17) Fan et al. (42), our core segmentation loss, *L*_seg_, is a hybrid loss composed of the sum of Binary Cross-Entropy Loss (*L*_BCE_) and Dice Loss (*L*_Dice_), as shown in Equation 13:

(13) $$L_{\text{seg}}=L_{\text{BCE}}+L_{\text{Dice}}$$

The *L*_BCE_ term enforces pixel-level correctness, while the *L*_Dice_ term improves performance on imbalanced classes by maximizing the spatial overlap between the predicted mask and the ground truth.

This deep supervision scheme ensures that the intermediate layers of the decoder are explicitly guided toward producing semantically correct feature maps. By enforcing consistency across multiple scales, the model learns to effectively align high-resolution details from shallower layers with the robust semantic context from deeper layers, leading to more accurate and coherent polyp segmentation.

## 4 Experiment and results

### 4.1 Datasets

To rigorously evaluate our proposed model, we curated a high-quality segmentation dataset named the PolypSegDataset. This dataset comprises 1,302 images containing a total of 1,342 meticulously annotated polyp instances. A key characteristic of this dataset is its strong focus on large, clinically significant polyps, which constitute the vast majority of the samples. Furthermore, the dataset is highly standardized, with all images sharing a uniform resolution of 560 × 480 pixels, which facilitates direct and fair model comparison. The high fidelity of the ground-truth masks makes this dataset particularly well-suited for evaluating boundary-level segmentation accuracy.

To provide a comprehensive overview of the dataset’s characteristics, we conducted a detailed statistical analysis, with the key statistics summarized in **Table 1**. Regarding the Polyp Area Distribution, the data is overwhelmingly dominated by large polyps (≥ 5000 pixels), which account for a striking 87.1% of all instances. In contrast, small polyps (*<* 1000 pixels) are nearly absent at only 0.2%. This distinct composition makes the PolypSegDataset an ideal benchmark for developing and testing models on well-developed, clinically significant lesions rather than incipient polyps.

**Table 1:** Statistical analysis of the polyp area distribution within the curated PolypSegDataset.

Metric	Category/statistic	Value/percentage
Polyp Size Distribution	Small (*<* 1000 pixels)	0.2%
	Medium (1000 − 5000 pixels)	12.7%
	Large (*>* 5000 pixels)	87.1%

**Figure 3:**
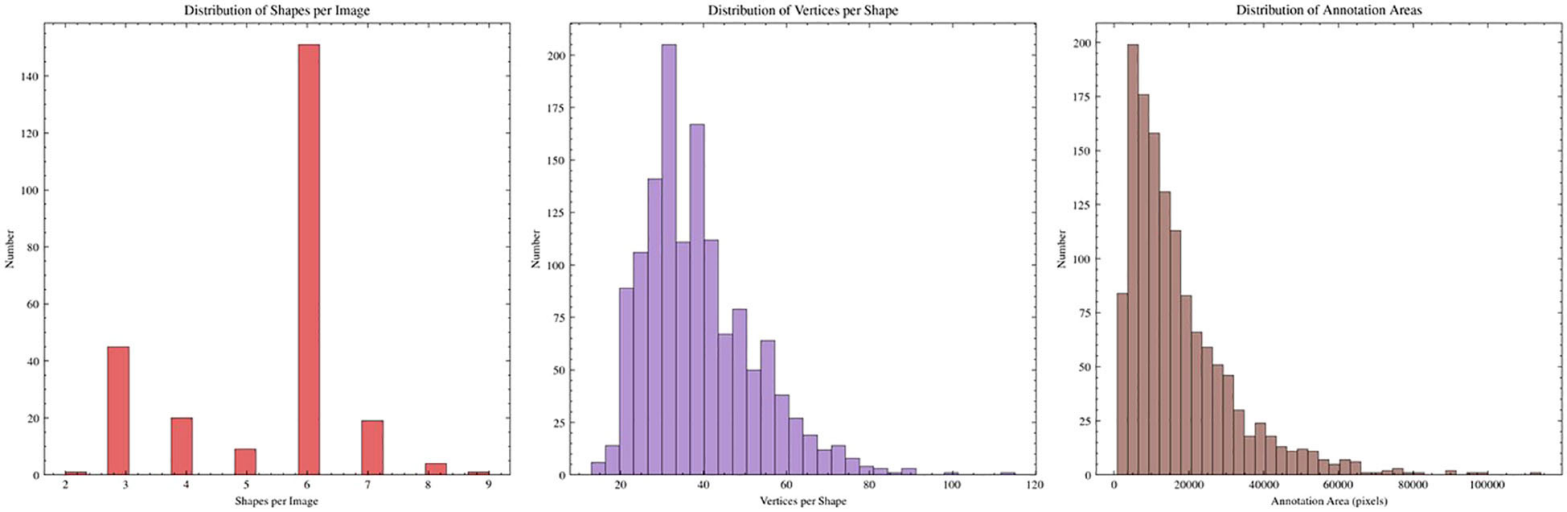
Analysis of the annotation precision in the PolypSegDataset, illustrated by the distribution of the number of vertices used per ground-truth mask. The data shows a high average of 38.79 vertices per annotation, which underscores the meticulous detail and high fidelity of the boundary tracing. This high-precision annotation makes the dataset particularly suitable for the rigorous evaluation of boundary-level segmentation accuracy.

Annotation Quality. **Figure 3** provides insight into the quality and precision of the ground-truth masks. The distribution of the number of vertices per annotation has a high average of 38.79. This metric underscores the meticulous detail with which the polyp boundaries were traced, moving far beyond simple bounding boxes or coarse outlines. Such high-precision annotation is critical for the rigorous evaluation of segmentation algorithms, especially for assessing boundary accuracy.

In addition to our curated PolypSegDataset, we also evaluate our model’s performance on two widely-used public benchmarks to ensure a comprehensive and fair comparison. We use CVC-ClinicDB Bernal et al. (56), a standard dataset containing 489 images with a variety of polyp types, and Kvasir-SEG Jha et al. (57), a larger and more diverse dataset consisting of 800 annotated images. Unlike our dataset, these public benchmarks feature a broader range of polyp sizes, shapes, and imaging conditions, allowing us to assess the generalizability and robustness of our proposed method.

### 4.2 Implementation details

We implement our proposed model using the PyTorch framework and conduct all experiments on a single NVIDIA 4090 GPU. The encoder backbone is initialized with the weights of SAM2-Hiera-Large pre-trained on the SA-1B dataset. For all three datasets—PolypSegDataset, CVC-ClinicDB, and Kvasir-SEG—we perform a stratified split into training (80%), validation (10%), and testing (10%) sets, using a fixed random seed of 42 to ensure the reproducibility of our results. The specific splits for the training, validation, and testing sets are 1041/130/131 for PolypSegDataset, 489/61/62 for CVC-ClinicDB, and 800/100/100 for Kvasir-SEG, respectively.

During the training phase, all input images are resized to a uniform resolution of 352 × 352 pixels. We train the model with a batch size of 12 for a total of 100 epochs. We employ the AdamW optimizer with an initial learning rate of 3 × 10^−4^ and a weight decay of 1 × 10^−5^. To ensure training stability, a learning rate warmup strategy is utilized for the first 20 epochs. Furthermore, to prevent overfitting and select the best-performing model checkpoint, we implement an early stopping mechanism that monitors the segmentation loss on the validation set.

### 4.3 Evaluation metrics and compared methods

To provide a comprehensive and multi-faceted evaluation of segmentation performance, we employ eight widely-used metrics: mean Dice Coefficient (mDice), mean Intersection over Union (mIoU), Mean Absolute Error (MAE), S-measure (*S_α_*), weighted F-measure $(F_{\beta }^{w}),$ mean E-measure $(mE_{\xi }),$ mean Sensitivity (meanSen), and mean Specificity (meanSpe).

Among these, mDice and mIoU are region-based similarity metrics that evaluate the overlap between the predicted mask and the ground truth, primarily assessing the overall accuracy of the segmented object. MAE offers a direct pixel-by-pixel comparison by calculating the average absolute difference between the continuous prediction map and the binary ground-truth mask. To evaluate the model’s classification performance on foreground and background pixels respectively, we report meanSen and meanSpe. Sensitivity measures the model’s ability to correctly identify polyp pixels (true positives), while Specificity measures its ability to correctly identify background pixels (true negatives). We also include three advanced metrics that capture more complex, human perception-aligned aspects of segmentation quality. The weighted $F_{\beta }^{w}$ balances precision and recall using non-uniform weights to better match visual assessment. The *S_α_* evaluates the structural similarity between the prediction and the ground truth at both the object and region levels. Finally, the $mE_{\xi }$ simultaneously captures both image-level statistics and local pixel-level matching.

To benchmark the performance of our proposed model, we conduct a comprehensive comparison against eight state-of-the-art and representative models from the field of semantic and medical image segmentation. The selected methods include classic architectures such as U-Net Ronneberger et al. (39), UNet++ Zhou et al. (40), ResUNet Zhang et al. (41), and DeepLabV3 Chen et al. (58), as well as more recent Transformer-based models like TransUNet Chen et al. (44). We also compare against several models designed specifically for polyp segmentation, including PraNet Fan et al. (42), Polyp-PVT Dong et al. (17), and CTNet Xiao et al. (59). For a fair and direct comparison, we utilize the publicly available, open-source implementations for all baseline methods.

### 4.4 Results

#### 4.4.1 Results analysis for Kvasir-SEG

The quantitative results of our model and eight other state-of-the-art methods on the Kvasir-SEG dataset are presented in **Table 2**. Our proposed method demonstrates superior performance, achieving the best results on five of the eight key evaluation metrics. Specifically, our model attains the highest scores in mIoU (85.03%), mDice (90.46%), meanSpe (98.40%), and weighted F-measure $\left(F_{\beta }^{w}\right)$ (89.53%), while also recording the lowest (best) MAE (0.0313). Compared to the strongest baseline, CTNet, our model shows a notable improvement in the critical region-based similarity metrics of mDice and mIoU. Furthermore, our method achieves highly competitive, second-best results on the structural similarity metrics *S_α_* (92.47%) and $mE_{\xi }$ (94.52%). While some methods like Polyp-PVT exhibit slightly higher sensitivity, our model’s leading specificity score indicates a robust ability to minimize false positives by correctly identifying background regions.

**Table 2:** Quantitative comparison of our proposed method against eight state-of-the-art models on the Kvasir-SEG test set.

Method	Publication	MAE↓	*S_α_*↑	$F_{\beta }^{w}$↑	*mE_ξ_*↑	meanSen↑	meanSpe↑	mDice↑	mIoU↑
U-Net	MICCAI 2015	0.0446	0.8820	0.8132	0.9024	0.8866	0.9739	0.8470	0.7735
UNet++	TMI 2019	0.0432	0.8869	0.8326	0.9116	0.8851	0.9761	0.8562	0.7871
DeepLab V3	CVPR 2017	0.0444	0.8826	0.8111	0.9096	0.8888	0.9718	0.8478	0.7667
ResUNet	CVPR 2016	0.0827	0.7808	0.6578	0.8262	0.7683	0.9579	0.7103	0.6029
PraNet	MICCAI 2020	0.0378	0.8915	0.8481	0.9171	0.8651	0.9829	0.8630	0.7950
Polyp-PVT	AIR 2023	0.0337	0.9076	0.8717	0.9368	0.9284	0.9774	0.8909	0.8296
TransUNet	MIA 2024	0.0361	0.9194	0.8691	0.9394	0.9166	0.9776	0.8992	0.8396
CTNet	TCYB 2024	0.0319	**0.9263**	0.8794	**0.9468**	**0.9305**	0.9767	0.8969	0.8412
Ours		**0.0313**	0.9247	**0.8953**	0.9452	0.9052	**0.9840**	**0.9046**	**0.8503**

We report performance across eight different evaluation metrics. The best results are highlighted in bold, and the second-best results are underlined.

**Figure 4:**
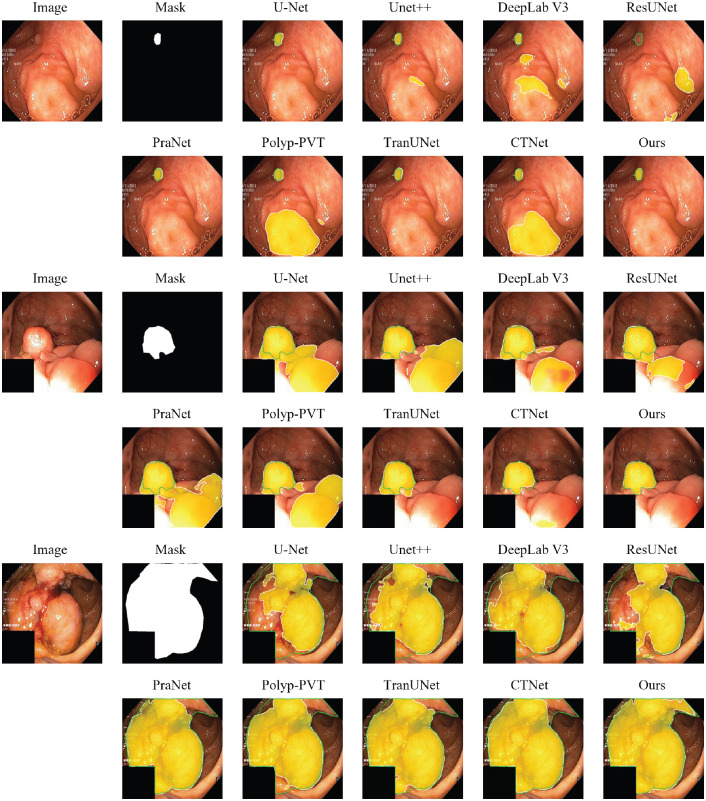
Qualitative comparison of segmentation results on Kvasir-SEG dataset. The green contour represents the ground-truth (GT) boundary. The model’s prediction is visualized as a semi-transparent yellow heatmap, where opacity indicates confidence. The final predicted boundary, after applying a 0.5 threshold, is shown as a white contour.

In addition to the quantitative metrics, **Figure 4** provides a qualitative comparison of the segmentation results from different models on representative images from the Kvasir-SEG test set. The visual results consistently illustrate that our model produces more precise and complete segmentation masks that more accurately adhere to the ground-truth boundaries. In challenging cases involving ambiguous edges, low contrast between the polyp and the surrounding mucosa, or irregular shapes, our method demonstrates enhanced robustness. It successfully maintains the integrity of the polyp structure while other methods, such as PraNet or U-Net, may yield incomplete masks or struggle with fine boundary details. This visual evidence corroborates the quantitative findings, highlighting our model’s improved ability to handle the complexities of polyp segmentation.

#### 4.4.2 CVC-ClinicDB

**Figure 5:**
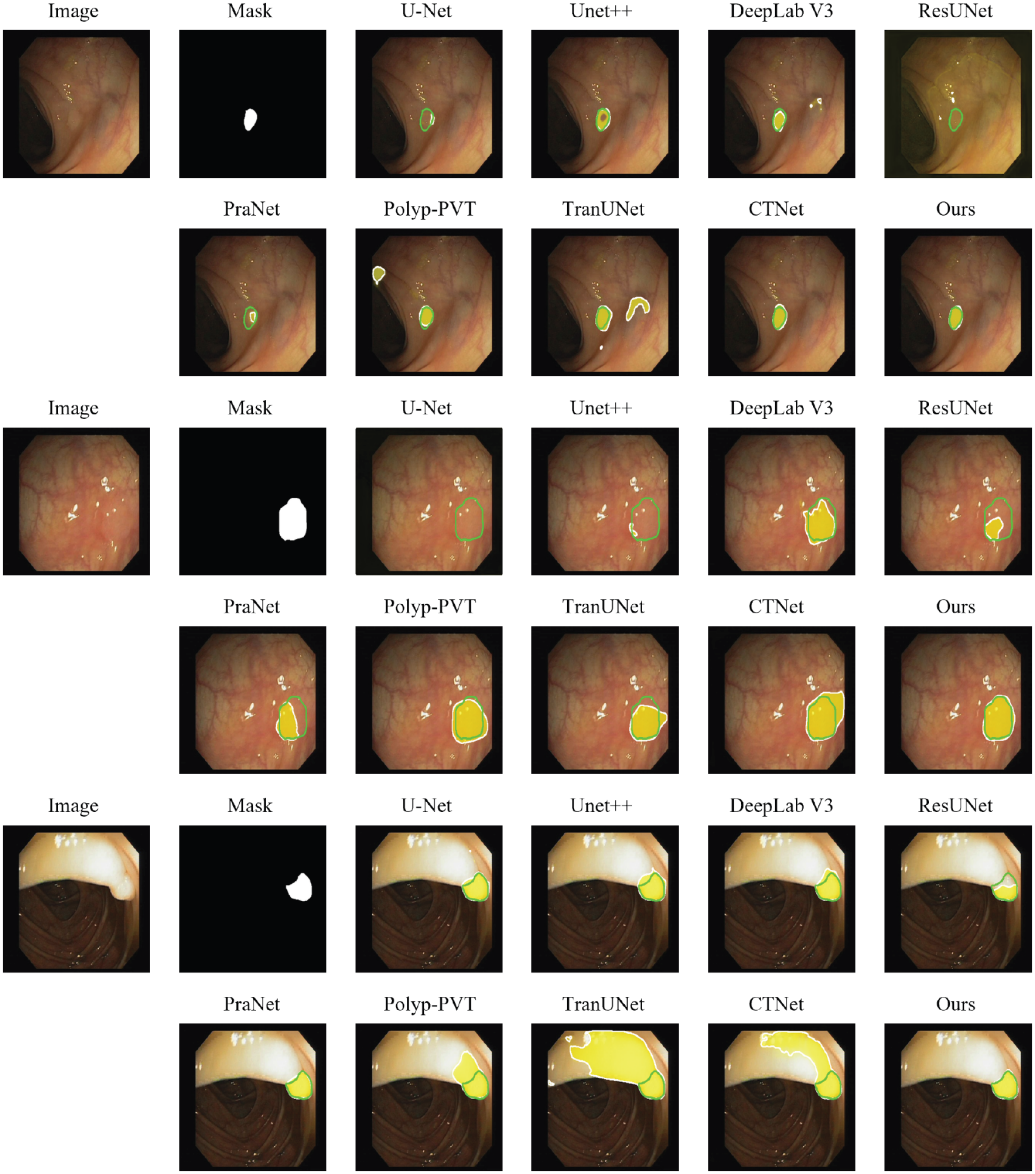
Qualitative comparison of segmentation results on CVC-ClinicDB dataset. The green contour represents the ground-truth (GT) boundary. The model’s prediction is visualized as a semi-transparent yellow heatmap, where opacity indicates confidence. The final predicted boundary, after applying a 0.5 threshold, is shown as a white contour.

**Table 3** presents the comparative results on the standard CVC-ClinicDB benchmark. On this highly competitive dataset, our model demonstrates state-of-the-art performance on the core metrics for segmentation accuracy. Specifically, our method achieves the highest mDice (93.16%) and mIoU (88.02%), which are the primary indicators of segmentation quality. It also records the best meanSpe (99.10%), highlighting its excellent ability to avoid false positives. While other methods like PraNet and Polyp-PVT show strong performance on certain perceptual metrics such as MAE and *S_α_*, our model’s superiority in mDice and mIoU suggests it produces the most spatially accurate and reliable segmentation masks overall. The qualitative results in **Figure 5** further support these findings, where our model consistently generates smooth and precise boundaries that closely adhere to the ground truth.

**Table 3:** Quantitative comparison of our proposed method against eight state-of-the-art models on the CVC-ClinicDB test set.

Method	Publication	MAE↓	*S_α_*↑	$F_{\beta }^{w}$↑	mEξ↑	meanSen↑	meanSpe↑	mDice↑	mIoU↑
U-Net	MICCAI 2015	0.0116	0.9348	0.9018	0.9575	0.9209	0.9899	0.9043	0.8540
UNet++	TMI 2019	0.0105	0.9418	0.9069	0.9679	0.9261	0.9900	0.9165	0.8663
DeepLab V3	CVPR 2017	0.0114	0.9412	0.9138	0.9755	0.9312	0.9902	0.9203	0.8610
ResUNet	CVPR 2016	0.0357	0.8580	0.7173	0.8989	0.8415	0.9696	0.7839	0.7054
PraNet	MICCAI 2020	**0.0093**	0.9532	0.9293	**0.9802**	0.9506	0.9909	0.9306	0.8724
Polyp-PVT	AIR 2023	0.0117	**0.9537**	**0.9295**	0.9703	0.9558	0.9898	0.9307	0.8687
TransUNet	MIA 2024	0.0131	0.9416	0.9002	0.9638	**0.9661**	0.9849	0.9126	0.8585
CTNet	TCYB 2024	0.0132	0.9525	0.8815	0.9726	0.9448	0.9855	0.9278	0.8727
Ours		0.0108	0.9461	0.9263	0.9773	0.9504	**0.9910**	**0.9316**	**0.8802**

We report performance across eight different evaluation metrics. The best results are highlighted in bold, and the second-best results are underlined.

#### 4.4.3 PolypSegDataset

**Figure 6:**
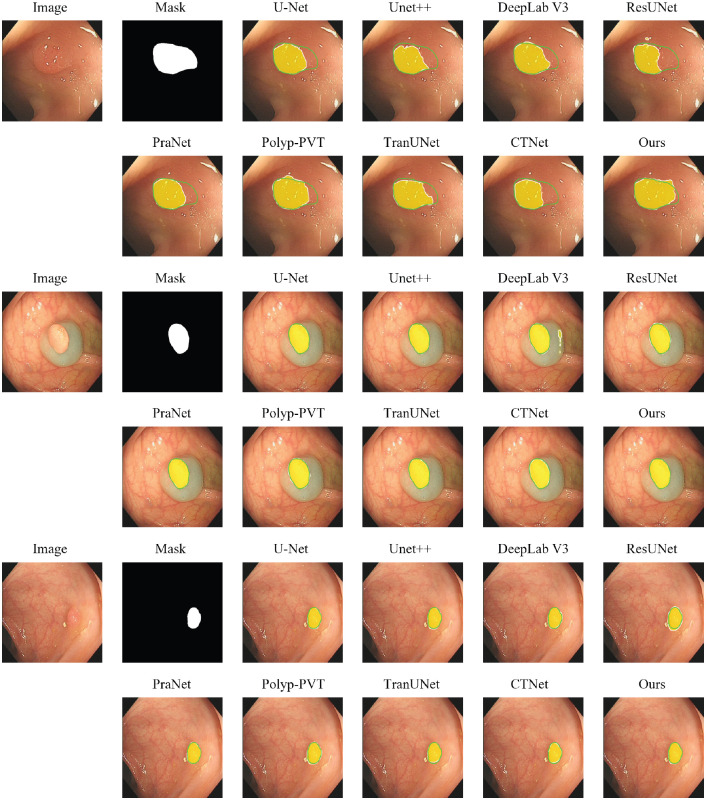
Qualitative comparison of segmentation results on PolypSegDataset. The green contour represents the ground-truth (GT) boundary. The model’s prediction is visualized as a semi-transparent yellow heatmap, where opacity indicates confidence. The final predicted boundary, after applying a 0.5 threshold, is shown as a white contour.

The performance of all methods on our curated PolypSegDataset is detailed in **Table 4**. The results demonstrate the clear superiority of our proposed model on this benchmark, as our method achieves state-of-the-art performance on seven out of the eight evaluation metrics, often by a significant margin. It secures the top scores for mIoU (88.62%), mDice (93.27%), meanSen (94.34%), all three perceptual metrics ( $S_{\alpha }$, $F_{\beta }^{w}$, and $mE_{\xi }$), and achieves the lowest MAE (0.0060). Given that PolypSegDataset is characterized by large polyps with high-quality boundary annotations, this strong performance validates our architecture’s effectiveness in precisely segmenting well-defined, clinically significant lesions. The qualitative examples provided in **Figure 6** corroborate these quantitative results, highlighting our model’s ability to generate exceptionally clean and accurate segmentation masks where the predicted contour almost perfectly overlaps with the ground-truth boundary.

**Table 4:** Quantitative comparison of our proposed method against eight state-of-the-art models on the PolypSegDataset test set.

Method	Publication	MAE↓	*S_α_*↑	$F_{\beta }^{w}$↑	mEξ↑	meanSen↑	meanSpe↑	mDice↑	mIoU↑
U-Net	MICCAI 2015	0.0095	0.9279	0.8751	0.9401	0.8837	0.9930	0.8793	0.8261
UNet++	TMI 2019	0.0090	0.9401	0.8994	0.9567	0.8993	0.9933	0.9004	0.8502
DeepLab V3	CVPR 2017	0.0089	0.9394	0.8937	0.9656	0.9120	0.9925	0.9036	0.8415
ResUNet	CVPR 2016	0.0272	0.8557	0.7394	0.8959	0.8265	0.9793	0.7741	0.6804
PraNet	MICCAI 2020	0.0066	0.9570	0.9272	0.9779	0.9336	0.9935	0.9203	0.8613
Polyp-PVT	AIR 2023	0.0083	0.9455	0.9046	0.9734	0.9339	0.9902	0.9201	0.8634
TransUNet	MIA 2024	0.0082	0.9506	0.9133	0.9748	0.9422	0.9917	0.9216	0.8690
CTNet	TCYB 2024	0.0074	0.9568	0.9219	0.9773	0.8982	**0.9940**	0.9221	0.8689
Ours		**0.0060**	**0.9606**	**0.9274**	**0.9788**	**0.9434**	0.9930	**0.9327**	**0.8862**

We report performance across eight different evaluation metrics. The best results are highlighted in bold, and the second-best results are underlined.

#### 4.4.4 Ablation study

To validate the effectiveness and individual contributions of our key proposed components, we conduct a comprehensive ablation study on the PolypSegDataset. We establish a strong baseline using the SAM2 encoder with our trainable adapter and the progressive refinement decoder. We then incrementally add our two main contributions: the Function-Specialized Mixture-of-Experts (FS-MoE) module and the Spectral-Guided Boundary Enhancement (SGBE) module. The results of this study are summarized in **Table 5**.

**Table 5:** Ablation study on the PolypSegDataset dataset.

Method	MAE↓	*S_α_*↑	$F_{\beta }^{w}$↑	*mE_ξ_*↑	meanSen↑	meanSpe↑	mDice↑	mIoU↑
SAM2+Adapter	0.0084	0.9463	0.9058	0.9687	0.9021	0.9892	0.9176	0.8558
+ FS-MoE Module	0.0079	0.9489	0.9127	0.9718	0.9264	0.9903	0.9229	0.8589
+ SGBE Module	0.0073	0.9498	0.9186	0.9751	0.9378	0.9917	0.9274	0.8611
SpetraNet	**0.0070**	**0.9504**	**0.9231**	**0.9768**	**0.9432**	**0.9930**	**0.9302**	**0.8627**

The table shows the performance improvement by progressively adding different modules to the baseline (SAM2+Adapter). The FS-MoE and SGBE modules both contribute to consistent gains across all metrics, and the full model (SpetraNet) achieves the best overall performance.

The best results are highlighted in bold.

As shown in the table, starting from the baseline, the integration of the FS-MoE module yields consistent performance gains across all eight metrics. This confirms the benefit of its adaptive, function-specialized feature refinement for handling diverse polyp appearances. Similarly, adding the SGBE module to the baseline also results in substantial improvements across the board, which highlights the critical role of frequency-domain enhancement in improving boundary definition and overall segmentation accuracy. Our full model, SpectraNet, which combines both modules, achieves the best performance on all metrics. The synergistic effect of spectral boundary enhancement and adaptive spatial refinement leads to the highest mIoU (86.27%) and mDice (93.02%) scores. This step-by-step analysis clearly demonstrates that both of our proposed modules are effective and contribute positively to the final performance of the network.

#### 4.4.5 Generalization capability evaluation

To rigorously assess the generalization capability of SpectraNet and ensure it does not overfit to specific data distributions, we conducted a cross-dataset evaluation. In this setting, we trained the model on a composite dataset merging Kvasir-SEG and CVC-ClinicDB, and then directly evaluated its performance on an unseen dataset: CVC-ColonDB Bernal et al. (60). This testing scenario is particularly challenging as CVC-ColonDB contains polyps with highly diverse appearances and imaging conditions distinct from the training set.

The quantitative results are summarized in **Table 6**. As observed, SpectraNet maintains robust performance on the unseen domain, achieving a mDice of 73.85% and a mIoU of 64.92%. Compared to state-of-the-art methods, our model demonstrates superior generalization ability, consistently outperforming representative methods such as Polyp-PVT and CTNet. This confirms that the proposed Spectral-Guided Boundary Enhancement (SGBE) and Function-Specialized Mixture-of-Experts (FS-MoE) modules facilitate the learning of intrinsic, invariant polyp features (such as structural phase information) rather than memorizing dataset-specific biases.

**Table 6:** Generalization performance evaluation.

Method	mDice	MIoU
U-Net	0.6425	0.5618
Polyp-PVT	0.6982	0.6105
CTNet	0.7156	0.6289
**SpectraNet (Ours)**	**0.7385**	**0.6492**

Models are trained on the combined Kvasir-SEG and CVC-ClinicDB datasets and tested on the unseen CVC-ColonDB dataset. The best results are highlighted in bold.

#### 4.4.6 Complexity analysis

To evaluate the computational efficiency of the proposed model, we compared SpectraNet with three representative state-of-the-art methods: PraNet, PolypPVT, and CTNet. The evaluation metrics include the total number of parameters (Total Params), the number of trainable parameters (Trainable Params), computational complexity (GFLOPs), and inference speed (Frames Per Second, FPS). All measurements were conducted on a single NVIDIA RTX 4090 GPU with an input resolution of 352 × 352.

The results are presented in **Table 7**. It is observed that SpectraNet has a significantly larger count of total parameters (214.90 M) and GFLOPs (125.076) compared to the lightweight baselines. This is expected as our architecture is built upon the heavy SAM2 foundation model to leverage its robust feature extraction capabilities. However, a key advantage of our design is the implementation of *Parameter-Efficient Fine-Tuning*. By freezing the heavy backbone and only training the lightweight adapters and decoder heads, SpectraNet requires only 2.75 M trainable parameters—reducing the training burden by over 90% compared to PraNet (32.55 M) and CTNet (44.29 M).

**Table 7:** Computational complexity analysis.

Model	Total params (M)	Trainable (M)	GFLOPs	FPS
PraNet	32.55	32.55	13.150	61.29
PolypPVT	25.11	25.11	10.018	67.93
CTNet	44.29	44.29	15.413	35.93
SpectraNet (Ours)	214.90	2.75	125.076	15.04

We compare the model parameters (Total and Trainable), computational cost (GFLOPs), and inference speed (FPS) with state-of-the-art methods. Note that SpectraNet utilizes a frozen backbone strategy, resulting in minimal trainable parameters.

In terms of inference speed, SpectraNet achieves 15.04 FPS. While this is lower than the lightweight models, it strikes a favorable trade-off between computational cost and the significant performance gains demonstrated in previous sections (e.g., +2-3% mDice). Furthermore, 15 FPS is generally considered sufficient to provide near real-time feedback in clinical colonoscopy workflows, where the priority is often the precision of the segmentation mask rather than ultra-high frame rates.

## 5 Conclusion

In this paper, we addressed the challenging problem of high-precision polyp segmentation, focusing on the critical issue of ambiguous boundary definition. We introduced SpectraNet, a novel encoder-decoder architecture that integrates a unique hybrid-domain enhancement strategy. Our approach leverages a Spectral-Guided Boundary Enhancement (SGBE) module to explicitly amplify structural details in the frequency domain, effectively sharpening the representation of polyp boundaries. This is complemented by a Function-Specialized Mixture-of-Experts (FS-MoE) module, which provides an adaptive mechanism to apply targeted feature refinement based on the specific characteristics of each polyp.

Comprehensive evaluations conducted across three distinct datasets confirmed the effectiveness of our design. On our curated, high-quality PolypSegDataset, as well as on the standard public benchmarks Kvasir-SEG and CVC-ClinicDB, SpectraNet consistently outperformed a suite of state-of-the-art segmentation models. The quantitative results highlighted our model’s superiority in achieving higher mIoU and mDice scores, while the qualitative comparisons demonstrated its ability to generate more accurate and complete masks with finer boundary details. The success of our approach validates the significant potential of integrating frequency-domain analysis and adaptive, function-specialized processing into deep learning frameworks for medical image segmentation. Future work may include extending this architecture to other medical imaging modalities where boundary ambiguity is a key challenge, such as tumor segmentation in MRI or CT scans, and exploring model compression techniques to facilitate real-time clinical application.

## Data Availability

The datasets presented in this article are not readily available because PolypSegDataset, was collected at Jiangyan Hospital Affiliated to Nanjing University of Chinese Medicine and is not publicly available due to ethical restrictions and patient privacy concerns outlined in the Institutional Review Board approval (Protocol No. 2025-008-003). A de-identified subset of PolypSegDataset supporting the findings of this study is available from the corresponding author, J.L. (Jing Ling), upon reasonable request. Requests to access the datasets should be directed to Jing Ling, 157770425@qq.com.

## References

[1] Center MM Jemal A Smith RA Ward E . Worldwide variations in colorectal cancer. CA.: A. Cancer J Clin. (2009) 59:366–78. doi: 10.3322/caac.20038, PMID: 19897840

[2] Ladabaum U Dominitz JA Kahi C Schoen RE . Strategies for colorectal cancer screening. Gastroenterology. (2020) 158:418–32. doi: 10.1053/j.gastro.2019.06.043, PMID: 31394083

[3] Brenner H Chang-Claude J Jansen L Seiler CM Hoffmeister M . Role of colonoscopy and polyp characteristics in colorectal cancer after colonoscopic polyp detection: a population-based case–control study. Ann Internal Med. (2012) 157:225–32. doi: 10.7326/0003-4819-157-4-201208210-00002, PMID: 22910933

[4] Miller SF Knight AR . The early detection of colorectal cancer. Cancer. (1977) 40:945–9. doi: 10.1002/1097-0142(197708)40:2<945::AID-CNCR2820400253>3.0.CO;2-F 890677

[5] Guachi L Guachi R Bini F Marinozzi F . Automatic colorectal segmentation with convolutional neural network. Comput-Aided. Des. Appl. (2019) 16. doi: 10.14733/cadaps.2019.836-845

[6] Viscaino M Bustos JT Munoz P Cheein CA Cheein FA . Artificial intelligence for the early detection of colorectal cancer: A comprehensive review of its advantages and misconceptions. World J Gastroenterol. (2021) 27:6399. doi: 10.3748/wjg.v27.i38.6399, PMID: 34720530 PMC8517786

[7] Jiaxing Z Hao T . Sam2 for image and video segmentation: A comprehensive survey. arXiv. (2025).

[8] Li S Ren Y Yu Y Jiang Q He X Li H . A survey of deep learning algorithms for colorectal polyp segmentation. Neurocomputing. (2025) 614:128767. doi: 10.1016/j.neucom.2024.128767

[9] Ji G-P Xiao G Chou Y-C Fan D-P Zhao K Chen G . Video polyp segmentation: A deep learning perspective. Mach Intell Res. (2022) 19:531–49. doi: 10.1007/s11633-022-1371-y

[10] Huo J Xiao R Zheng H Liu Y Ourselin S Sparks R . (2024). Matchseg: Towards better segmentation via reference image matching, in: Proceedings of the 2024 IEEE International Conference on Bioinformatics and Biomedicine (BIBM), pp. 2068–73. Piscataway: IEEE.

[11] Gupta M Mishra A . A systematic review of deep learning based image segmentation to detect polyp. Artif Intell Rev. (2024) 57:7. doi: 10.1007/s10462-023-10621-1

[12] Qayoom A Xie J Ali H . Polyp segmentation in medical imaging: challenges, approaches and future directions. Artif Intell Rev. (2025) 58:169. doi: 10.1007/s10462-025-11173-2

[13] Akbari M Mohrekesh M Nasr-Esfahani E Soroushmehr SR Karimi N Samavi S . (2018). Polyp segmentation in colonoscopy images using fully convolutional network, in: Proceedings of the 40th Annual International Conference of the IEEE Engineering in Medicine and Biology Society (EMBC), Piscataway. pp. 69–72. IEEE., PMID: 10.1109/EMBC.2018.8512197 30440343

[14] Yeung M Sala E Schönlieb C-B Rundo L . Focus u-net: A novel dual attention-gated cnn for polyp segmentation during colonoscopy. Comput Biol Med. (2021) 137:104815. doi: 10.1016/j.compbiomed.2021.104815, PMID: 34507156 PMC8505797

[15] Sun X Zhang P Wang D Cao Y Liu B . (2019). Colorectal polyp segmentation by u-net with dilation convolution, in: Proceedings of the 18th IEEE International Conference on Machine Learning and Applications (ICMLA), Piscataway. pp. 851–8. IEEE.

[16] Duc NT Oanh NT Thuy NT Triet TM Dinh VS . Colonformer: An efficient transformer based method for colon polyp segmentation. IEEE Access. (2022) 10:80575–86. doi: 10.1109/ACCESS.2022.3195241

[17] Dong B Wang W Fan D-P Li J Fu H Shao L . Polyp-pvt: Polyp segmentation with pyramid vision transformers. arXiv. (2021).

[18] Jha D Tomar NK Sharma V Bagci U . Transnetr: transformer-based residual network for polyp segmentation with multi-center out-of-distribution testing. In: Medical Imaging with Deep Learning. PMLR (2024). p. 1372–84.

[19] Shao H Zhang Y Hou Q . (2024). Polyper: Boundary sensitive polyp segmentation, in: Proceedings of the AAAI conference on artificial intelligence, , Vol. 38. Palo Alto, CA: AAAI Press. pp. 4731–9.

[20] Mei J Zhou T Huang K Zhang Y Zhou Y Wu Y . A survey on deep learning for polyp segmentation: Techniques, challenges and future trends. Visual Intell. (2025) 3:1. doi: 10.1007/s44267-024-00071-w

[21] Liu Z Zheng S Sun X Zhu Z Zhao Y Yang X . The devil is in the boundary: Boundary-enhanced polyp segmentation. IEEE Trans Circuits. Syst Vid. Technol. (2024) 34:5414–23.

[22] Tajbakhsh N Chi C Gurudu SR Liang J . (2014). Automatic polyp detection from learned boundaries, in: Proceedings of the 2014 IEEE 11th International Symposium on Biomedical Imaging (ISBI), Piscataway: IEEE. pp. 97–100.

[23] Bracewell RN . The fourier transform. Sci Am. (1989) 260:86–95. doi: 10.1038/scientificamerican0689-86, PMID: 2727659

[24] Shanmugam KS Dickey FM Green JA . An optimal frequency domain filter for edge detection in digital pictures. IEEE Trans Pattern Anal Mach Intell. (1979), 37–49. doi: 10.1109/TPAMI.1979.4766874, PMID: 21868829

[25] Nawab H Oppenheim A Lim J . (1981). Improved spectral subtraction for signal restoration, in: Proceedings of the IEEE International Conference on Acoustics, Speech, and Signal Processing (ICASSP ’81), Piscataway: IEEE. Vol. 6. pp. 1105–8.

[26] Pogorelov K Ostroukhova O Jeppsson M Espeland H Griwodz C De Lange T . (2018). Deep learning and hand-crafted feature based approaches for polyp detection in medical videos, in: Proceedings of the 2018 IEEE 31st International Symposium on Computer-Based Medical Systems (CBMS), Piscataway: IEEE. pp. 381–6.

[27] Mamonov AV Figueiredo IN Figueiredo PN Tsai Y-HR . Automated polyp detection in colon capsule endoscopy. IEEE Trans Med Imaging. (2014) 33:1488–502. doi: 10.1109/TMI.2014.2314959, PMID: 24710829

[28] Maghsoudi OH . Superpixel based segmentation and classification of polyps in wireless capsule endoscopy. In: 2017 IEEE Signal Processing in Medicine and Biology Symposium (SPMB). Piscataway, NJ: IEEE (2017). p. 1–4.

[29] Rahim T Usman MA Shin SY . A survey on contemporary computer-aided tumor, polyp, and ulcer detection methods in wireless capsule endoscopy imaging. Comput. Med Imaging Graphics. (2020) 85:101767. doi: 10.1016/j.compmedimag.2020.101767, PMID: 32966967

[30] Brandao P Zisimopoulos O Mazomenos E Ciuti G Bernal J Visentini-Scarzanella M . Towards a computed-aided diagnosis system in colonoscopy: automatic polyp segmentation using convolution neural networks. J Med Robot. Res. (2018) 3:1840002. doi: 10.1142/S2424905X18400020

[31] He K Zhang X Ren S Sun J . (2016). Deep residual learning for image recognition, in: Proceedings of the IEEE conference on computer vision and pattern recognition, Piscataway, NJ: IEEE. pp. 770–8.

[32] Simonyan K Zisserman A . Very deep convolutional networks for large-scale image recognition. arXiv. (2014).

[33] Cai L Wu M Chen L Bai W Yang M Lyu S . (2022). Using guided self-attention with local information for polyp segmentation, in: Lecture Notes in Computer Science (LNCS), International Conference on Medical Image Computing and Computer-Assisted Intervention (MICCAI 2022), Cham: Springer. pp. 629–38.

[34] Tomar NK Jha D Bagci U Ali S . (2022). Tganet: Text-guided attention for improved polyp segmentation, in: Lecture Notes in Computer Science (LNCS), International Conference on Medical Image Computing and Computer-Assisted Intervention (MICCAI 2022), Cham: Springer. pp. 151–60., PMID: 10.1007/978-3-031-16437-8_15 PMC9912908 36780239

[35] Zhang R Lai P Wan X Fan D-J Gao F Wu X-J . (2022). Lesion-aware dynamic kernel for polyp segmentation, in: Lecture Notes in Computer Science (LNCS), International Conference on Medical Image Computing and Computer-Assisted Intervention (MICCAI 2015), Cham: Springer. pp. 99–109.

[36] Sengar SS Meulengracht C Boesen MP Overgaard AF Gudbergsen H Nybing JD . Multi-planar 3d knee mri segmentation via unet inspired architectures. Int J Imaging Syst Technol. (2023) 33:985–98. doi: 10.1002/ima.22836

[37] Singh O Sengar SS . Betternet: An efficient cnn architecture with residual learning and attention for precision polyp segmentation. arXiv. (2024).

[38] Khan M Fu S Ullah I . Attention-guided asymmetric multiscale polyp segmentation network. IEEE Trans Instrum. Meas. (2025). doi: 10.1109/TIM.2025.3550626

[39] Ronneberger O Fischer P Brox T . (2015). U-net: Convolutional networks for biomedical image segmentation, in: International Conference on Medical Image Computing and Computer-Assisted Intervention (MICCAI 2020), Cham: Springer. pp. 234–41.

[40] Zhou Z Rahman Siddiquee MM Tajbakhsh N Liang J . Unet++: A nested u-net architecture for medical image segmentation. In: International workshop on deep learning in medical image analysis. Cham: Springer (2018). p. 3–11., PMID: 10.1007/978-3-030-00889-5_1 PMC7329239 32613207

[41] Zhang Z Liu Q Wang Y . Road extraction by deep residual u-net. IEEE Geosci Remote Sens Lett. (2018) 15:749–53. doi: 10.1109/LGRS.2018.2802944

[42] Fan D-P Ji G-P Zhou T Chen G Fu H Shen J . (2020). Pranet: Parallel reverse attention network for polyp segmentation, in: International conference on medical image computing and computer-assisted intervention, Cham: Springer. pp. 263–73.

[43] Dosovitskiy A Beyer L Kolesnikov A Weissenborn D Zhai X Unterthiner T . An image is worth 16x16 words: Transformers for image recognition at scale. arXiv. (2020).

[44] Chen J Mei J Li X Lu Y Yu Q Wei Q . Transunet: Rethinking the u-net architecture design for medical image segmentation through the lens of transformers. Med Img. Anal. (2024) 97:103280. doi: 10.1016/j.media.2024.103280, PMID: 39096845

[45] Khan M Ullah I Khan N Hussain S Khattak MI . Adpnet: Attention-driven dual-path network for automated polyp segmentation in colonoscopy. Img. Vision Comput. (2025), 105648. doi: 10.1016/j.imavis.2025.105648

[46] Vaswani A Shazeer N Parmar N Uszkoreit J Jones L Gomez AN . Attention is all you need. Adv Neural Inf Process Syst. (2017) 30.

[47] Kirillov A Mintun E Ravi N Mao H Rolland C Gustafson L . (2023). Segment anything, in: Proceedings of the IEEE/CVF international conference on computer vision, Piscataway, NJ: IEEE. pp. 4015–26.

[48] Li H Zhang D Yao J Han L Li Z Han J . (2024). Asps: Augmented segment anything model for polyp segmentation, in: International Conference on Medical Image Computing and Computer-Assisted Intervention (MICCAI 2024), Cham: Springer. pp. 118–28.

[49] Li Y Hu M Yang X . Polyp-sam: Transfer sam for polyp segmentation. In: Medical imaging 2024: computer-aided diagnosis, vol. 12927. Bellingham, WA: SPIE (2024). p. 749–54.

[50] Ravi N Gabeur V Hu Y-T Hu R Ryali C Ma T . Sam 2: Segment anything in images and videos. arXiv. (2024).

[51] Ryali C Hu Y-T Bolya D Wei C Fan H Huang P-Y . Hiera: A hierarchical vision transformer without the bells-and-whistles. In: ICML (PMLR) (2023). PMLR p. 29441–54.

[52] Houlsby N Giurgiu A Jastrzebski S Morrone B De Laroussilhe Q Gesmundo A . Parameter-efficient transfer learning for nlp. In: ICML (PMLR) (2019). PMLR p. 2790–9.

[53] Qiu Z Hu Y Li H Liu J . Learnable ophthalmology sam. arXiv. (2023).

[54] Zhou Y Lei T Liu H Du N Huang Y Zhao V . Mixture-of-experts with expert choice routing. Adv Neural Inf Process Syst. (2022) 35:7103–14.

[55] Kittler J . On the accuracy of the sobel edge detector. Img. Vision Comput. (1983) 1:37–42.

[56] Bernal J Sánchez FJ Fernández-Esparrach G Gil D Rodríguez C Vilariño F . Wm-dova maps for accurate polyp highlighting in colonoscopy: Validation vs. saliency maps from physicians. Comput. Med Imaging Graphics. (2015) 43:99–111. doi: 10.1016/j.compmedimag.2015.02.007, PMID: 25863519

[57] Jha D Smedsrud PH Riegler MA Halvorsen P De Lange T Johansen D . (2019). Kvasir-seg: A segmented polyp dataset, in: Lecture Notes in Computer Science (LNCS), International Conference on Multimedia Modeling (MMM 2019), Cham: Springer. pp. 451–62.

[58] Chen L-C Papandreou G Schroff F Adam H . Rethinking atrous convolution for semantic image segmentation. arXiv. (2017).

[59] Xiao B Hu J Li W Pun C-M Bi X . Ctnet: Contrastive transformer network for polyp segmentation. IEEE Trans Cybern. (2024) 54:5040–53., PMID: 38470573 10.1109/TCYB.2024.3368154

[60] Bernal J Sánchez J Vilarino F . Towards automatic polyp detection with a polyp appearance model. Pattern Recognit. (2012) 45:3166–82.

